# Comparison of the Microleakages of Four Root-End Filling Materials: An In Vitro Study

**DOI:** 10.7759/cureus.40461

**Published:** 2023-06-15

**Authors:** Angitha S, Saleem Azhar, Rishi Manan, Neetu Bansal, Digvijay Singh, Bharat Chauhan

**Affiliations:** 1 Conservative Dentistry and Endodontics, Institute of Dental Studies & Technologies, Ghaziabad, IND

**Keywords:** root canal treatment, endodontics, totalfill bioceramic root repair material, retrograde filling, apical microleakage

## Abstract

Introduction: When a nonsurgical endodontic treatment is ineffective, surgery is necessary. This entails putting a retrofilling to seal the tooth’s apex. Exposing the lesion, performing a curettage, exposing the root apex, resecting it, preparing the root end, and lastly filling the cavity with the proper material are all steps in endodontic surgery. Thus, the aim of this study is to compare the apical microleakage of four root-end filling materials in cavities prepared using ultrasonic retro tip in in vitro conditions.

Materials and Methods: An in vitro study was conducted on 60 extracted single-rooted teeth and was cut at the cementoenamel junction (CEJ). They were biomechanically prepared and obturated. Apical 3 mm root-end resection was done using a diamond disc. Root-end cavities were made using an ultrasonic retro tip. Teeth were separated into four groups and filled with SuperEBA^®️^ ethoxy-benzoic acid (EBA; Keystone Industries, New Jersey), mineral trioxide aggregate (MTA), Biodentine (Septodont, France), and TotalFill Bioceramic Root Repair Material (BC RRM; FKG Dentaire Sàrl, Switzerland). The samples were kept in methylene blue dye and split longitudinally. The degree of dye penetration was observed under a stereomicroscope and scored. Finally, the results were analyzed.

Results: TotalFill BC RRM and Biodentine showed the least apical microleakage (p <0.05). Group 1 samples had the highest mean microleakage, followed by Group 2, Group 3, and Group 4 samples.

Conclusion:All of the sample groups showed some evidence of microleakage, but not all of the samples showed leaking. SuperEBA (Group 1) demonstrated the highest microleakage when compared to the other groups.

## Introduction

A root canal treatment is the most commonly employed treatment to disinfect and fill the root canal system three-dimensionally. However, sometimes, despite thorough chemomechanical preparation and obturation, orthograde treatment may fail [1]. Periapical surgery is indicated when there is excessive root canal calcification, separated instruments extending beyond the apex, iatrogenic perforations, ledges or shoulder, and teeth restored with crowns or post and core. It may also be used in symptomatic cases that have not responded to conventional root canal treatment [2]. Periradicular surgery includes debridement and curettage, root apex exposure and resection, and retrograde cavity preparation followed by appropriate filling material insertion [3]. In addition, after a 1-year postoperative follow-up, the success rate of periapical surgery for patients with periapical lesions was 73.9% [4]. One of the typical clinical conditions affecting the periradicular tissues are periapical lesions [5]. One of the most commonly performed procedures for surgical endodontic treatment is retrograde obturation, which uses a variety of techniques and materials [6].

A retrograde filling is important to create an apical seal that prevents the microleakage of remaining irritants into periradicular tissues [7]. The prime factor that impacts a successful periapical surgery is the selection of retrograde filling material. Initially, amalgam, silver cones, gold foil, gutta-percha (GP), composite resins, zinc oxide eugenol (ZOE) cement, polycarboxylate cement, zinc phosphate cement, glass ionomer cement (GIC), and titanium screws were used for the same [8]. SuperEBA^®️^ ethoxy-benzoic acid (EBA) Cement (Keystone Industries, New Jersey) is a type of reinforced ZOE cement with 68% EBA and 32% eugenol. ZOE cement as a root-end filling material was unsuccessful; nevertheless, the reinforced variant showed better results [9]. Mineral trioxide aggregate is considered an ideal retrograde material [1]. Mineral trioxide aggregate (MTA) possesses significant qualities, such a high pH, biocompatibility, fixing power despite humidity, periradicular regeneration, and osteoinductive ability [10]. Biodentine (Septodont, France) is calcium silicate-based cement with high biocompatibility. It shows better physical and chemical properties, such as reduced time for setting and increased mechanical properties that make it a compatible root-end filling material [11]. A reparative biocompatible substance that seals perforations and does not irritate surrounding tissues is required in cases of extensive perforation [12].

TotalFill Bioceramic Root Repair Material (BC RRM; FKG Dentaire Sàrl, Switzerland) is a premixed material either in putty or syringe form. Its main components are calcium silicates and zirconium oxide. This material showed superior healing in periradicular surgery. It sets fast and shows improved handling properties. In addition, biocompatibility is analogous to MTA [13]. Apical seals attained through a retrograde filling material can be evaluated by the depth of the dye penetration, fluid-filtration technique, radioisotope or bacteria penetration, or electrochemical methods. The dye penetration technique is the prevalent and easily performed method [14]. The fluid filtration technique evaluates the endodontic and restorative sealers’ capacity for sealing. As a result, this method has established credibility in the field of study evaluating apical and coronal microleakage. Because of this, compared to the dye method, the fluid filtration method relied on quantitative measurements of fluid passage within the interfaces; both procedures produced results that were comparable in earlier studies [15]. To date, no in vitro studies have been conducted to compare and evaluate apical microleakage after using SuperEBA, MTA, Biodentine, and TotalFill BC RRM in cavities prepared using ultrasonic retro tips [16,17]. The present in vitro study aims to compare the apical microleakage of four root-end filling materials in cavities prepared using ultrasonic retro tips and evaluate the results. The null hypothesis was that there would be no significant difference in the apical microleakage amid the four chosen materials.

## Materials and methods

The sample size was calculated using the formula *n *= *Z^2^ P* (1−*P*) *d^2^*, where *n *is the sample size, *Z *is the statistic corresponding to the level of confidence, and *P *is the expected prevalence. A power of 0.80 with an alpha (α) level of 0.05 (confidence level = 95%), and a sample size of 60 was considered for the total samples. A total of 60 single-rooted extracted teeth, which were periodontally compromised and indicated for extraction, were collected from the Department of Oral & Maxillofacial Surgery, IDST. Soft tissues and deposits were mechanically removed from all the samples using Gracey curettes, and teeth were inspected under a stereomicroscope microscope (Carl Zeiss, Jena, Germany) for examining the number of canals, cracks/defects, and decay. The specimens were stored in 10% formalin until use.

Teeth with single root and single canal, closed apex, without fractures, resorption, or cracks were included. Multirooted teeth and teeth with extra canals, open apex, root caries, and calcification were excluded. The coronal part of the teeth was sectioned horizontally along the long axis with a diamond disc, at the cementoenamel junction (CEJ) level or below, to standardize the root length (15 mm).

Pre-operative radiography was performed, and access openings were created with an access bur (Dentsply Maillefer, USA). The working length was estimated radiographically with a #10 K-file, and a #40 K-file was used as the master apical file (Mani Inc., Japan). Ethylenediaminetetraacetic acid (EDTA) liquid irrigation was performed first (Prevest DenPro, India), and then 5% sodium hypochlorite irrigation was performed (Prevest DenPro, India), followed by saline irrigation. The canals were dried and obturated with lateral compaction technique using 2% gutta-percha (GP) cones (Meta Biomed, Korea) and AH Plus (Dentsply Maillefer, Ballaigues, Switzerland) as the root canal sealer.

Following obturation, cavities were filled with composite resin. The treated teeth were kept in saline for one week. Then, teeth were kept in an incubator (Binder, Tuttlingen, Germany), which has a specification of 535 L of interior space with a footprint of just 0.58 m2 at 100% humidity at 37 ℃ for five days. The apical 3 mm was cut using a straight fissure bur. Class I cavity was prepared in the root end with an ultrasonic retro tip to a depth of 3 mm. The samples were randomly divided into four groups with 15 teeth each: Group 1 (N = 15) retrograde cavity filled with SuperEBA, Group 2 (N = 15) with MTA, Group 3 (N = 15) with Biodentine, and Group 4 (N = 15) with TotalFill BC RRM.

**Figure 1:**
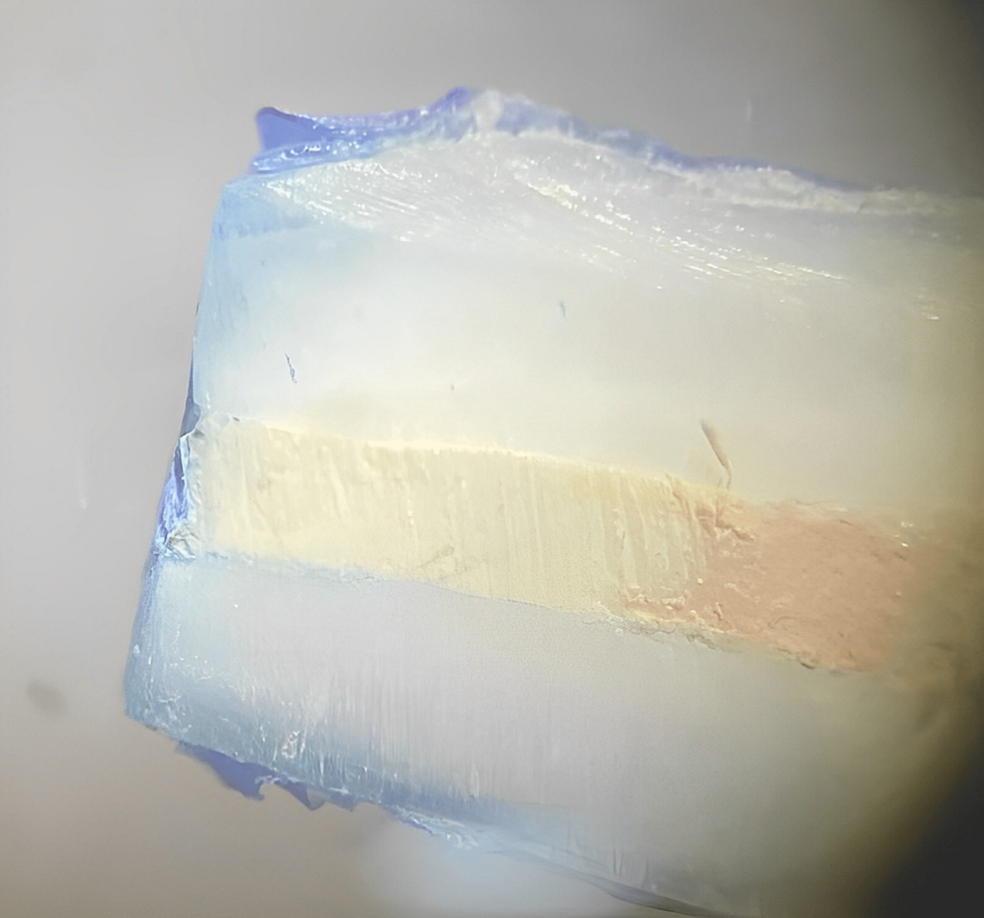
Stereomicroscopic image of the setup tested

The materials were manipulated according to the manufacturers’ instructions, followed by filling the cavities. The teeth were stored in 100% humidity at 37 ℃ for five days. The prepared retro-cavities underwent cleaning, saline irrigation, and drying. Then, they were coated with nail varnish, except for the apical 1 mm, and dried. Next, the teeth were placed in 1% methylene blue dye for up to 48 h. The roots were washed and split into longitudinal sections along the long axis using a diamond disc. Dye penetration was examined under a stereomicroscope, and the scoring was done on a scale of 0 to 4 (Figure 1).

This in vitro study was conducted in the Department of Conservative Dentistry and Endodontics at Institute of Dental Studies & Technologies (IDST), Kadrabad, Modinagar, Ghaziabad, Uttar Pradesh, India. Ethical clearance was obtained from the Institutional Review Board (IRB) with IRB number IDST/IEC/2019/PG/11.

Statistical analysis

Data were analyzed with IBM SPSS Statistics for Windows, Version 21 (Released 2012; IBM Corp., Armonk, New York, United States. As the main outcome variable was ordinal, non-parametric tests, such as Kruskal-Wallis and Mann-Whitney U tests, were utilized for the analysis.

## Results

Table 1 shows the description of microleakage scores among all the study groups.

**Table 1:** Description of the microleakage scores among all the study groups

Microleakage score					
	N (number)	Mean (M)	Standard deviation	95% confidence interval for the mean	
				Lower bound	Upper bound
Group 1	15	1.4000	0.63246	1.0498	1.7502
Group 2	15	0.8667	0.51640	0.5807	1.1526
Group 3	15	0.4000	0.50709	0.1192	0.6808
Group 4	15	0.2000	0.41404	-0.0293	0.4293

Table 2 shows the post-hoc pairwise comparison by the Mann-Whitney U test.

**Table 2:** Post-hoc pairwise comparison by the Mann-Whitney U test

Comparisons	P-values of the pairwise comparison by the Mann-Whitney U test
Group 1 vs. Group 2	0.019
Group 1 vs. Group 3	<0.001
Group 1 vs. Group 4	<0.001
Group 2 vs. Group 3	0.022
Group 2 vs. Group 4	0.001
Group 3 vs. Group 4	0.240

The findings indicate that microleakages in Group 3 and Group 4 samples were significantly lesser than in those in Group 2 samples and were further significantly lower than those in Group 1 samples. No significant difference could be found between Group 3 and Group 4 samples. The mean microleakage of Group 1 samples was maximum, followed by Group 2, Group 3, and Group 4 samples.

## Discussion

The accomplishment of periapical surgery is highly dependent on a proper apical seal. Retrograde filling materials are proposed to limit or avoid leakage into periapical tissues [15]. This study intended to test the microleakage of four retrograde materials. Ultrasonic retro tips are better than burs for retro preparation. The preparation of root-end cavity with ultrasonic tips causes negligible destruction to the root canal morphology. They are precise, conservative, and cleaner. The cutting bevel is 90° to the long axis of the root, which reduces the number of patent dentinal tubules at the open end and minimizes microleakage [18].

Ishikawa et al. assessed the retrograde cavity preparation with US retro tip and concluded that the use of US retro tip reduced the time taken in root-end cavity preparation [19]. The resulting cavities were more precise, and the US tips were more efficient in cutting than conventional burs. In this study, stainless-steel ultrasonic tip with diamond coating was used. The angle of root-end resection affects the leakage, so a 90° resection angle was selected here as it is considered more acceptable by previous studies. A resection depth of 3 mm reduces the lateral canals by 93% and apical ramifications by 98% [20].

The most commonly used method to evaluate the sealing property of retrograde materials is dye leakage. It can provide an estimate of the sealing property in various clinical conditions. In the present study, the linear penetration of 1% methylene blue dye was measured. Methylene blue is commonly used because of its small molecular weight that aids in penetrability [21]. The disadvantage of this method is that the dye molecules are smaller in size than bacteria, which can result in an overestimation of the bacteria’s penetration levels due to microleakage [20]. Lucena-Martin et al. showed that the transverse root section method results in the loss of dye and dentine portion [22]. Consequently, the longitudinal sectioning method was performed here to evaluate dye penetration into filling materials.

Epoxy resin-based AH Plus sealer shows high flowability, and it can penetrate dentinal tubules at deeper levels. Increased polymerization boosts the interlocking of the sealer material and dentin [23]. Therefore, in this study, AH Plus root canal sealer was chosen for obturation. Of all the materials used for retrograde filling, the least microleakage was observed for TotalFill BC RRM (Group 4), followed by Biodentine (Group 3), MTA (Group 2), and SuperEBA (Group 1). SuperEBA is broadly studied for retrograde fillings, and it has shown satisfactory properties. Greer et al. and Suntimuntanakul et al. observed that SuperEBA EBA Cement shows a higher sealing property when compared to a few other retrograde filling materials [24]. The results of our study are in agreement with other studies in which MTA showed improved marginal seal than other retrograde filling materials, such as GIC, amalgam, light cure GIC, and SuperEBA [25]. This can be because of the hydroxyapatite-like crystal formation at material-root canal dentine interfaces, which results in excellent adhesion preventing the penetration of the dye [26]. MTA has always shown less leakage than SuperEBA; there was no or minimal dye leakage in the majority of MTA specimens [27]. Similarly, Bates et al. traced microleakage in dental amalgam, SuperEBA, and MTA and concluded that it was the least in MTA [28]. Biodentine shows superior characteristics to MTA as it sets faster, thus reducing the risk of bacterial contamination [29]. Biodentine exhibits superior sealing properties than MTA [30]. In addition, it exhibits better biomineralization than MTA, with broader calcium-rich layer formation. Radeva et al. also found similar results as this study; they concluded that Biodentine shows greater sealing ability than MTA [31].

TotalFill BC RRM is available in the premixed syringe delivery system or putty form. It is extremely resistant to washout. It has calcium phosphate monobasic as an additional agent that enhances hydroxyapatite formation. It shows properties, such as wear resistance, biocompatibility, chemical durability, and aesthetics [32].

Nonetheless, this study has some limitations. Instead of employing a scanning electron microscope (SEM) or confocal microscope, which may have provided significantly more information and reliable results, the investigation was conducted with a rather limited sample size. The research used only a few materials other materials are not tested, so it could be a limitation. The materials used in the study also will have limitations regarding their properties and hence in the future, a long-term trial could be done on various other materials. As a result, additional research including more samples is needed.

## Conclusions

Although not all of the samples had leakages, all of the sample groups displayed some degree of microleakage. When compared to the other groups, SuperEBA demonstrated the highest level of microleakage. Comparing the two materials to the other materials, TotalFill BC RRM and Biodentine demonstrated the least amount of apical microleakage. Therefore, it is possible to suggest using these materials. A larger sample size should be used in in vivo experiments in the future to study the interactions between the sealing cement that were used, as well as the therapeutic significance and consequences of solubility over time.
